# Unpacking response Inhibition in animals – part 2: an empirical test

**DOI:** 10.1007/s10071-025-02033-1

**Published:** 2026-01-28

**Authors:** Camille A. Troisi, Alizée Vernouillet, Reinoud Allaert, Sophia Knoch, An Martel, Luc Lens, Frederick Verbruggen

**Affiliations:** 1 Centre for Research on Ecology, Cognition and Behaviour of Birds, Ghent University, Ghent, Belgium; 2 Department of Experimental Psychology, Ghent University, Ghent, Belgium; 3 Université de Rennes, Normandie Université, CNRS, EthoS (Ethologie animale et humaine), UMR 6552, Rennes, 35000 France; 4 Department of Biology, Ghent University, Ghent, Belgium; 5 Department of Pathobiology, Pharmacology and Zoological Medicine, Ghent University, Ghent, Belgium

**Keywords:** Inhibitory control, Response inhibition, Task context, Herring gulls, Lesser black-backed gulls

## Abstract

Response inhibition - the ability to suppress or stop actions - is crucial for adaptive behaviour across species. In Part 1 of this study, we presented a conceptual framework for understanding response inhibition as multifaceted. Specifically, we conceptualised response inhibition as a race between a ‘go runner’ and a ‘stop runner’. These runners are modulated by the type of stimulus that triggers the runner, the relative timing between stimuli, and the type of actions that are elicited. In Part 2, the framework is used to make predictions about correlations between different measures of response inhibition across three tasks: the detour barrier task, the thwarting task, and the stop-change task. These predictions were tested in two closely related bird species: herring gulls (*Larus argentatus*) and lesser black-backed gulls (*Larus fuscus*). The correlations between specific measures of response inhibition were generally weak. This pattern of results supports the idea that response inhibition is not a unitary ability, but rather a set of partly independent components. These findings highlight the importance of task context and trigger type in shaping inhibitory performance, and they illustrate how conceptual and mathematical models can guide more nuanced interpretations of inhibition across different ecological and experimental settings.

**Supplementary Information:**

The online version contains supplementary material available at 10.1007/s10071-025-02033-1.

## Introduction

Response inhibition - the ability to suppress or stop actions - is a cornerstone of adaptive behaviour in animals, allowing them to respond flexibly to changing environments and avoid costly mistakes (e.g., Ashton et al. 2018; Coomes et al. 2022). For example, a bird preparing to forage must inhibit its approach if a predator suddenly appears, demonstrating the critical role of response inhibition in survival and decision-making. Similarly, animals must often resist the urge to consume food that is perceived but inaccessible, such as the smell or sight of fruit in hard shells, until they have found a way to access it (e.g. by cracking the shell). Social interactions also require response inhibition (e.g., Gobbo and Zupan Šemrov 2022; Overduin-de Vries et al. 2024); for example, animals must suppress aggressive responses to stronger opponents during territorial disputes or courtship to avoid unnecessary injury. These diverse scenarios highlight the ecological and evolutionary importance of response inhibition in navigating complex environments.

Despite its complexity, response inhibition is often treated as a unidimensional psychological ‘ability’, with tasks and measures assumed to be interchangeable across studies and species (see Beran 2015). In Part 1 of this study, we challenged this ‘unidimensionality’ assumption by proposing a conceptual framework for understanding response inhibition. Drawing on insights from cognitive psychology and neuroscience, we conceptualised response inhibition as a race between two ‘runners’: a ‘go runner’, triggered by a ‘go’ stimulus that prompts action, and a ‘stop runner’, triggered by stimuli that signal inhibition (Logan and Cowan 1984; Verbruggen and Logan 2009). Furthermore, our framework highlights how variability in stimulus type, timing, and the nature of the action being inhibited modulates these runners in the race (Troisi et al. 2026). This task-specific perspective may be essential for disentangling the cognitive and non-cognitive factors that influence performance, providing a more nuanced understanding of response inhibition. For a detailed discussion of the framework, its theoretical underpinnings, and its application to common tasks, we refer readers to Part 1 (Troisi et al. 2026).

In this second part, we use the conceptual framework to test empirical predictions about correlations between measures of response inhibition across three of the tasks analysed in Part 1: the detour barrier, thwarting, and stop-change tasks. The detour barrier task was selected because it is, along with its many variants, one of the most commonly used paradigms in animal cognition research for assessing response inhibition (see Kabadayi et al. 2018 for a review). In this task, individuals encounter a transparent barrier with a visible reward—typically food or, in some social variants, conspecifics—positioned behind it. To succeed, they must resist the impulse to move directly toward the reward and instead navigate around the barrier. In most studies, success on the detour task is measured as the number of trials in which individuals avoid touching the barrier and detour directly. However, individuals may also obtain the reward after initially bumping into the barrier and then adjusting their behaviour. The task thus provides insight into the inhibition of both a discrete action (going straight) and a repetitive action (repeated interactions with the barrier), which are not entirely independent. Therefore, to more cleanly disentangle these components, we included two additional tasks that allowed us to independently assess the inhibition of discrete versus repetitive actions: the thwarting task and the stop-change task.

In the thwarting task, a visible but inaccessible reward (typically food) is placed beneath a transparent lid in a test box. Because the reward cannot be obtained, individuals must inhibit repetitive attempts to access it, such as pecking, pawing, or biting at the lid. The optimal response is thus to stop interacting with the food bowl and disengage. This makes the task well-suited for assessing the inhibition of persistent, ineffective actions (e.g. Lucon-Xiccato et al. 2020). In the stop-change task, the reward is initially visible in one location within a test box. As the individual approaches, the reward shifts—typically via a mechanism (e.g., a seesaw) that hides the original reward while revealing it at a new location. To succeed, individuals must inhibit a discrete, prepotent action—approaching the original reward location—and quickly initiate a new action toward the updated location. This task thus provides a clear measure of the inhibition of discrete motor responses. It was explicitly developed with the race model in mind and allows for direct application of that framework (see Meier et al. 2017, 2020 for studies using this task in birds).

Together, these three tasks allowed us to probe response inhibition across different task contexts. Using the common conceptual framework, we then made predictions about between-task correlations in behavioural measures. Specifically, we first examined whether measures related to the ‘go runner’—that is, individuals’ initial response to the food stimulus—were correlated across tasks. In all three tasks (detour barrier, thwarting, and stop-change), the ‘go runner’ is triggered by the presentation of food, and each task involves some form of approach behaviour toward this reward. We therefore expected measures of ‘going’ (see Methods for details) to show positive correlations across tasks. Such inter-individual consistency could reflect underlying traits such as general processing speed, motivational state, or broader personality dimensions like activity and exploration (Carere and Locurto 2011; Dougherty and Guillette 2018; Sih and Del Giudice 2012; Troisi et al. 2019).

**Fig. 1:**
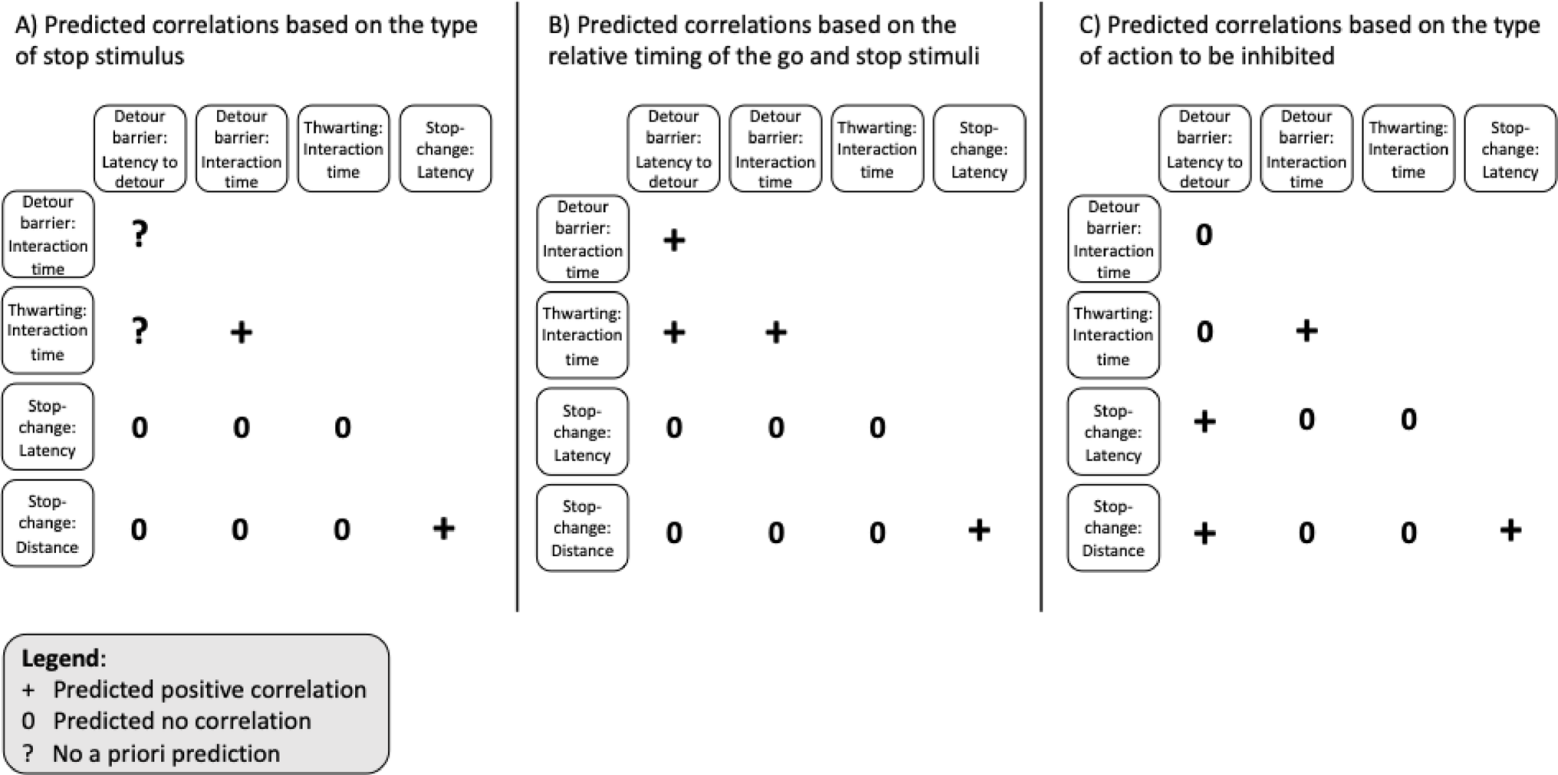
Predicted correlations between different measures of ‘stopping’ (top row and first column; see main text for description) based on the overlap between the type of ‘stop stimulus’ (external vs. internal) (**A**), the relative timing of ‘go’ and ‘stop’ stimuli (**B**), and the type of action that has to be stopped (repetitive vs. discrete) (**C**). + indicates that we predicted a correlation; (0) indicates that we predicted no correlation. Note that we could not always make a priori predictions for the detour barrier task, (indicated by?), as the initial ‘stop stimulus’ in this task is unclear (see Table 1); however, once they pecked at the barrier, the ‘stop stimulus’ in the detour barrier task would be similar to the ‘stop stimulus’ in the thwarting task (i.e. a transparent object or the failure to obtain a reward). Measures of ‘going’ are not included in this figure, but correlations between them across tasks were predicted. We did not make any predictions about correlations between measures of ‘going’ and ‘stopping’

While the ‘go’ runner was similar across tasks, the tasks were deliberately selected to capture variation in how response inhibition is triggered and expressed. Specifically, the tasks differ in the nature of the ‘stop’ stimulus, the timing between ‘go’ and ‘stop’ stimuli, and the type of action that must be inhibited (Table 1). As a result, we did not expect uniform correlations for the ‘stop runner’ across all measures. Instead, we predicted that correlations would emerge selectively, depending on the degree of overlap in task features (see Fig. 1; for a detailed task analysis, see also Part 1: Troisi et al. 2026). In the stop-change task, we can directly measure the stop-change latency (the latency between the time the bird triggers the seesaw, and the time it changes direction), as well as the birds’ distance from the unrewarded location. In the detour barrier task, we used the latency to successfully complete the detour as a measure of inhibition of the response to go straight (although this measure is again less pure than that obtained in the stop-change task, as ‘going’ and ‘stopping’ cannot be disentangled; see Part 1 for a more detailed discussion). In both the detour barrier and the thwarting task, we also measured the time individuals spend pecking at the transparent barrier.

First, in terms of ‘stop stimuli’ and their relative timing, the detour barrier and thwarting tasks are more similar to each other than to the stop-change task: both tasks have similar external and/or internal ‘stop stimuli’ (i.e. transparent objects and no reward, respectively), and there is presumably no delay between the presentation of the ‘go’ and ‘stop’ stimuli. In contrast, the stop-change task uses a different external ‘stop’ stimulus (a seesaw) that appears well after the ‘go’ stimulus (and after the ‘go runner’ has already been initiated). We therefore predicted that if the timing and nature of the ‘stop stimulus’ are key determinants of inhibitory control, then stop measures should correlate more strongly between the detour and thwarting tasks than with the stop-change task (see Tables 1 and Fig. 1A, B).

Second, in terms of the nature of the actions to be inhibited, the detour barrier and stop-change tasks both involve the inhibition of a discrete single action (i.e., running towards the food). If the type of action to be inhibited is an important determinant of performance, we would expect these measures to correlate across the two tasks (see Table 1; Fig. 1C). Furthermore, if inhibiting the initial response in the detour barrier task fails, it also measures the inhibition of a repetitive action (persisting interaction with the barrier), akin to the time the bird spends interacting with the (covered) food bowl during the thwarting task. We therefore predicted positive correlations between time spent pecking at the transparent barrier/cover in those two tasks (see Table 1; Fig. 1C).

**Table 1:** Task description, possible external and internal ‘stop stimuli’, relative timing of the ‘go’ and ‘stop’ stimuli, and type of action to be inhibited (with the corresponding dependent variables) for the three response Inhibition tasks. For all tasks the ‘go’ stimulus is the presence of food. We provide a more detailed task-based analysis in part 1 of this study

Task	Task description	External stop stimulus	Internal stop stimulus	Relative timing stimuli	Dependent variable(s) for inhibiting a discrete action	Dependent variable(s) for inhibiting a repetitive action
Detour barrier task	Inhibiting a predominant response (‘going’ straight towards the food), and if that fails, inhibiting pecking at a barrier	Transparent barrier, task context	Lack of reward, retrieval of previous detour memories	Go and Stop simultaneous	Latency to detour	Time spent interacting with the barrier
Thwarting task	Inhibiting an unrewarded behaviour (pecking at transparent cover/attempting to reach food)	Transparent cover	Lack of reward	Go and Stop simultaneous	N.A.	Time spent interacting with the apparatus
Stop-change task	Inhibiting a trajectory towards a no-longer rewarded food patch, and approach a novel food patch instead	Seesaw (covering old location and making new location available)	N.A.	Go before Stop	(1) Latency to change direction (2) Closest distance to the old location	N.A.

We tested our predictions in two closely related bird species—herring gulls (*Larus argentatus*) and lesser black-backed gulls (*Larus fuscus*). The two species are roughly similar in size, breed at the same time of year, lay an average of three eggs per season (Burger et al. 2020; Weseloh et al. 2020), and have comparable flight capacities (Camphuysen 1995). Both species are social, nesting in mixed-species colonies (Garthe et al. 1999), and are considered generalists, relying on marine, agricultural, and urban food sources (Camphuysen 1994; Garthe et al. 1999; Kim and Monaghan 2006; Sotillo et al. 2014). In recent decades (approximately the last 60 years), both species have colonised and adapted to urban environments for breeding and feeding (Belant 1997; Rock and Vaughan 2013; Spelt et al. 2019).

These species were chosen because they are semi-precocial: the chicks can be raised without parental care, allowing us to control and standardise their early-life environment (the effect of which is explored in separate study: Troisi et al. 2024). In addition, both are known for their high behavioural flexibility, both within and between individuals (Belant 1997; Rock and Vaughan 2013; Spelt et al. 2019, 2021; Tyson et al. 2015). For example, lesser black-backed gulls that specialise in fishing discards flexibly switch to alternative food sources on weekends when discards are unavailable (Tyson et al. 2015). Similarly, herring gulls adjust their foraging behaviour to human activity patterns, visiting schools during break times and waste centres on weekdays when food waste is more abundant (Spelt et al. 2021). This behavioural flexibility, combined with marked inter-individual variation in foraging strategies, makes these species a suitable model for studying response inhibition across different task contexts. Given the ecological and behavioural similarities outlined above, we did not expect species-level differences in the relationships between response inhibition measures, and therefore analysed all data from both species together.

## Methods

### Subjects

From May 2021 to June 2021, eggs of herring gulls and lesser black-backed gulls were collected on the Belgian coast (De Panne, Oostende, Blankenberge, Zeebrugge, Knokke). All individuals were hand-reared from egg. After hatching, chicks were first kept indoors and then moved to outdoor enclosures (10 m2) when they were approximately 5 days old. Each enclosure held 15 chicks of similar age (except for the last two enclosures where individuals had up to 13 days of age difference). Further information on egg collection, incubation, rearing and feeding can be found in the Supplementary Materials.

120 juvenile gulls (46 herring gulls and 74 lesser black-backed gulls; 61 females, 58 males, 1 unknown; see Table S1 for a further breakdown by species and sex) participated in our experiment. Species ID and sex were confirmed through DNA sampling, from down feathers collected on the day of hatching. If DNA sampling was not possible for an individual, we identified their species using morphological characteristics when they were ringed and predicted their sex with a support vector machine classifier using morphological data (see Supplementary Materials for a validation of this method).

Birds were fed under two feeding regimes as part of a separate study (see Supplementary Materials for more details on the feeding treatments). We did not expect the feeding regime to influence the relationship between the different ‘stop’ measures.

### Behavioural tests

#### Group habituation and training in the home enclosure

There were two feeding stations per enclosure, in which food was placed behind opaque barriers (see Figure S1 in Supplementary Materials). These opaque barriers were present throughout the experiment. This provided chicks with detour experience. In addition, three transparent barriers (50 × 100 cm width x height) were placed within the non-feeding area of the enclosure. These transparent barriers were present from the first day chicks were in the outside enclosure until the first day of testing and provided chicks with experience with transparency. Both the transparent and opaque barriers had coloured tape on the sides to delimit the area of the barrier. Learning may be required both to perform a successful detour to access food (Kabadayi et al. 2018) and to grasp the concept of transparent objects (van Horik et al. 2018). Therefore, we ensured that the necessary learning of the detour behaviour to access food, and of transparency, occurred prior to testing, so that the test trials would primarily reflect response inhibition rather than initial learning about transparency (Kabadayi et al. 2018). This procedure was intended to ensure that the barriers would function effectively as both external and internal ‘stop stimuli’ during testing (Troisi et al. 2026).

### General testing protocol

**Fig. 2:**
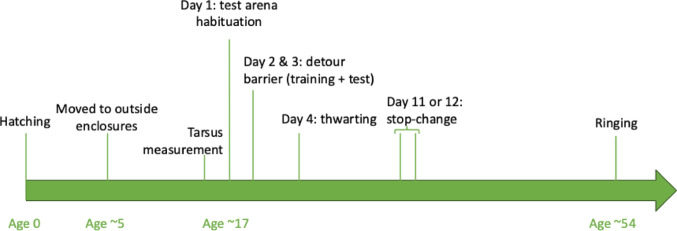
Timeline of the experiment, including mean age of the birds in days. As soon as individuals were moved to outside enclosures, they had experience with opaque and transparent barriers (transparent barriers were present until the first day of testing)

Behavioural tests started 7 days after the group was complete. See Fig. 2 for an overview. Mean age on the first day of testing was 16.7 days (range 13–21 days; due to human error, the exact hatching date was unknown for 19 individuals). The order of testing (detour barrier (training and testing), thwarting, and stop-change) was the same for all individuals, as this provides more power to study inter-individual variation.

Two enclosures (of similarly aged chicks) were tested each day (simultaneously). For each testing day, birds were food deprived at 18:00 the previous day and were tested in the morning (8:00–11:00). They were then fed after all birds from the enclosure had finished testing. For birds that did not eat during the task, this meant a maximum of 3 h delay in feeding, compared to birds that did eat during the task. Due to human error, the birds of one enclosure received food prior to testing on the 7th July 2021 (stop-change task), while on 16th July 2021 (also stop-change task), birds of another enclosure were not food deprived in the evening (but were not given food in the morning). Note that the stop-change task took place a few days after the other tests for practical reasons.

**Fig. 3:**
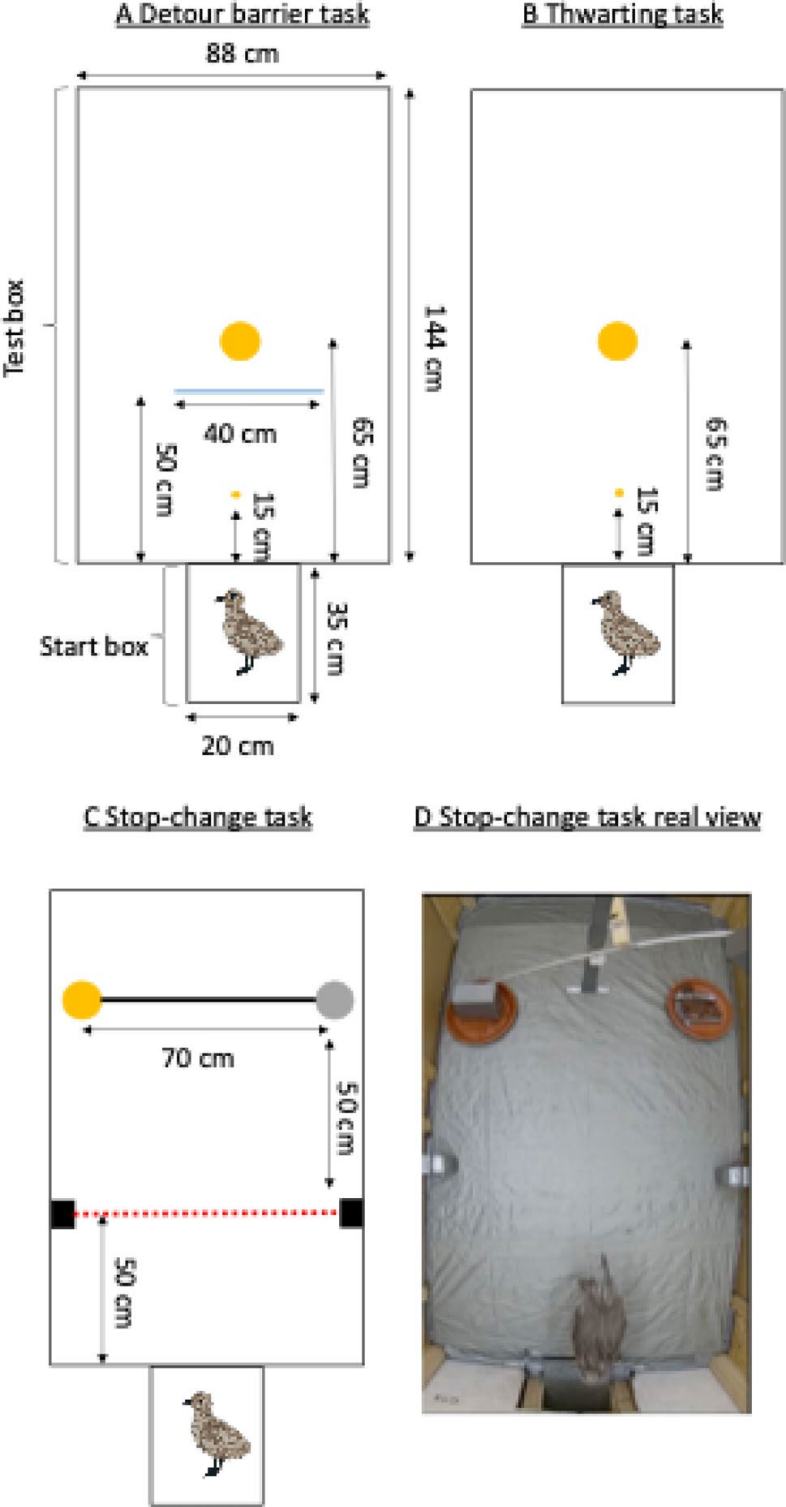
Schematics of the three tasks used, including dimensions (height start box: 26 cm, test box: 132 cm): (**A**) detour barrier task, (**B**) thwarting task, (**C**) stop-change task. The large yellow circles represent food, and the grey circle in the stop-change task represents food that is made non-available during the task. The small yellow circles (in the detour barrier and thwarting task) represent the start food (see main text). The blue line in the detour barrier task represents the (opaque or transparent) barrier. The red dotted line in the stop-change task represents the infrared beam. Gull drawings by AV (CC-BY 4.0). (**D**) is a real view of the stop-change task at the start of the trial, with the food on the left being covered, and the food on the right being initially accessible. See videos of all three tests in OSF: https://osf.io/3nbze/?view_only=d062486e8a9447d1a9262e06e9c0d989

Behavioural tests were conducted in two identical ‘test units’ (except for the side doors which were reversed), equipped with cameras which filmed trials from above. Each test unit consisted of a start box attached to a test box (dimensions in Fig. 3). Each enclosure was tested in one of our two identical test boxes, so each bird only experienced one test box (except during the stop-change task, which was done in only one test box, as each enclosure was tested on a separate day). Individuals were picked up by human experimenters from their enclosure and put in a cat carrier to transport them to the test boxes (max 20 m of transport). Six different experimenters were involved in handling the birds. They were then placed in a start box connected with a sliding door to the main test box (Fig. 3). This start box was included to standardise the start position and to allow individuals to voluntarily enter the test box. After a fixed interval (30 s in the detour and thwarting task; 15 s in the stop-change task), the door between the start box and the test box was opened to allow individuals to enter the test box. If birds did not enter the test box voluntarily (after 60 s in the detour and thwarting tasks; 30 s in the stop-change task), they were gently pushed forward. Trials ended either when individuals reached the food (for the detour barrier and stop-change tasks) or when the time limit of the trial was reached (detour & thwarting: 180 s; stop-change: 120 s). As the start boxes and test boxes were entirely closed, individuals could not see the experimenter during testing. At the end of the trial, individuals were caught, put back in their cat carrier, and placed in a dark room, to avoid further disturbance for the birds yet to be tested. The order of testing was semi-random: experimenters picked the first bird that they came across within the enclosure. Once all individuals finished testing, they were all placed back in their home enclosure and fed.

### Individual habituation in the test box

On Day 1, chicks were individually habituated to the test box. A food bowl (diameter: 17 cm) containing fish was placed 50 cm directly in front of the entrance. Enough food was placed to cover the bottom of the bowl, and for the food to be visible from the entrance. The same food bowl and same amount of food were used for the detour and thwarting tasks. A small piece of fish was also placed in front of the start box entrance (henceforth ‘start food’; 15 cm from the entrance) to encourage the birds to come out. Once the door between the start box and test box was opened, birds were left for 300 s in the test box.

### Detour barrier task

On Day 2 (individual detour training), an opaque barrier (40 * 40 cm length * height, made of cardboard, with coloured tape on each side delimiting the barrier) was placed 50 cm directly in front of the start box entrance, in front of the food bowl that was placed 65 cm from the start box entrance (see Fig. 3A). Start food was again placed at the entrance of the test box to encourage the birds to leave the start box. Once the door between the start box and test box was opened, birds were given a maximum of 180 s to detour the barrier and eat the food placed behind the barrier. On Day 3 (detour test), a similar set up to the individual training was used, except the barrier was made of transparent plastic. The trial stopped once the individual had eaten, or after 180 s after the start of the trial.

### Thwarting task

On Day 4, individuals took part in the thwarting task. The food bowl, covered by a transparent plastic sheet (rendering the food visible but inaccessible), was placed 65 cm from the entrance of the start box. The sheet – made of Plexiglas – was attached to the bowl with Velcro, so that the birds could not remove it. A piece of food was placed both 15 cm from the entrance and on top of the covered food bowl to ensure that birds interacted with the setup at least once, thereby eliciting a clear go response (pecking) that would subsequently need to be inhibited (Fig. 3B). After opening the door between the start box and the test box, individuals had 180 s to interact with the inaccessible food. After 180 s, the transparent cover over the food bowl was removed by the experimenter (by detaching the Velcro), making the food accessible. Individuals had 60 s to interact with the now-accessible food.

### Stop-change task

On Days 11 or 12 (depending on the enclosure), the stop-change task took place. We only tested one enclosure per day on the stop-change task, due to time constraints (and each bird was tested only once, like in the other tasks). The apparatus consisted of a seesaw with two cups, and an infrared beam triggering the seesaw. The infrared beam was placed 50 cm from the entrance of the start box, and the food bowls (with cups) were placed 50 cm from this beam (Fig. 3C). The food was visible from the entrance. At the start of the trial, the cup on the right was approximately 50 cm above the food bowl, while the cup on the left was covering the food. Food bowls were 70 cm apart (Fig. 3C and D). The seesaw was held in place by an electromagnet. Upon breaking the infrared beam, the magnet would switch off, allowing the seesaw to tilt, covering the food on the right (henceforth “old location”), and uncovering the food on the left (henceforth “new location”). Unlike in the other tasks, the start box had a transparent door, allowing individuals to directly see where the food was located before the start of the trial (always to the right, Fig. 3D). This allowed individuals to rapidly move towards the food from the start of the trial, as they already had the opportunity (while in the start box) to identify the location of the food, and this helped us identify the change of direction more easily. The birds were placed in the start box for 15 s, after which the door was opened. If individuals did not exit after 30 s, they were gently pushed forward into the test box. Those durations are shorter than in the other tasks, because birds could already see the food reward from the start box. The trial stopped once the individual had eaten some food, or 120 s after they had entered the test box.

### Dependent variables and inclusion criteria

We recorded all trials from above and videos were subsequently coded using BORIS (Friard and Gamba 2016). The behaviours measured are described in the following two sections. The video coder was naive to the hypotheses, treatment, and to the species and sex of the individual. To assess inter-rater reliability, 20% of videos were coded by a second coder (naïve to the species, treatment, and sex of the individual, but not naïve to the hypotheses). This was done to ensure that coders using the same ethogram produced consistent, objective and replicable data.

### The go runner (‘going’ measures)

In the detour barrier and thwarting tasks, we placed a small piece of food (‘start food’, see Fig. 3) at a distance of 15 cm from the start box to motivate the birds to enter the test box. For both tasks, the measure of ‘going’ was defined as the time difference between the moment of leaving the location of the start food (regardless of whether they ate the start food or not) and the moment of the first interaction with the task. For the detour barrier task, the first interaction was defined as either (a) the moment the bird made physical contact with the barrier (i.e. touching, usually by pecking) or (b) the moment its feet crossed the side of the barrier in cases where it detoured directly without interacting with it before eating the food behind the barrier. For the thwarting task, the first interaction was defined as either (a) a peck at the transparent cover itself or (b) a peck at the uncovered piece of fish placed on the bowl. As the food was placed directly on the apparatus, we considered pecking it a valid interaction with the setup. For the stop-change task, we used the time between leaving the start box (i.e. when the chick’s feet were outside the box) and the crossing of the infrared beam as our measure of ‘going’ (there was no start food in this task).

### The stop runner (‘stopping’ measures)

In the detour barrier task, we had two ‘stopping’ measures. First, for the inhibition of discrete actions, we used the latency to detour, which was the time between the moment the bird left the location of the start food and the time at which the bird’s feet crossed the side of the barrier. Second, for the inhibition of repetitive actions, we measured the time spent physically interacting with the barrier (i.e. pecking the barrier). For the thwarting task, we also measured the time spent physically interacting with the (covered) bowl (i.e. pecking the cover). These two measures also equate to continued execution of an unrewarded behaviour (i.e. persistence). Finally, for the stop-change task, we used the stop-change latency as our primary measure of ‘stopping’. This was defined as the time between the crossing of the infrared beam and the moment the chick changed direction (i.e., when it aligned the body to the new location instead of the old location). In line with previous work (Meier et al. 2017), we also used the smallest distance between the bird and the old location within a trial (i.e. the bird’s proximity to the old location) as a secondary measure. This distance was measured from video recording using software ‘Tracker’ (Brown et al. 2012) to calculate the distance, in pixels, between the bird and the old location (covered bowl) in each frame.

In the detour barrier task, the measures of ‘going’ and ‘stopping’ were the same (i.e. detour latency) if the individual did not interact with the barrier (i.e. ‘successfully’ detoured). We reran the analyses after excluding those individuals (see Supplementary Materials, Table S2).

### Inclusion criteria

In the analyses reported below, we only included birds that ‘participated’ in the tests (Table S1). We included this criterion to ensure that only data from birds that showed some motivation for the task were included (e.g. birds that spent the whole trial in a corner were excluded). In the detour barrier task, task participation was defined as interacting with the barrier or eating food behind the barrier (*n* = 99); in the thwarting task, this was defined as interacting with the covered food bowl or eating food once the cover was removed (*n* = 105); and in the stop-change task, this was defined as crossing the infrared beam (*n* = 105). We had to omit 7 individuals from the stop-change task because of technical issues, resulting in a sample size of 98 for this task.

### Ethical statement

We performed the experiment in accordance with the Association for the Study of Animal Behaviour ethical guidelines under permission of the ethical committee of animal experimentation (VIB Site Ghent, Universiteit Gent; EC2021-017). Eggs were collected under permit ANB/BL-FF/V21-00154.

### Use of AI

We used Large Language Models for proofreading (ChatGPT (versions 4 and 4o) and DeepL (free version)).

### Data Availability

Data and R Code are available on OSF: https://osf.io/jbe4q/?view_only=cd763b15f4b649fb80e520fea326f0a3.

### Statistical analysis

All analyses were conducted in R version 4.2.0 (R Core Team 2022). Inter-coder reliability was assessed using the interclass correlation coefficient from the icc function in the irr package (version 0.84.1, Gamer et al. 2019); consensus between the two coders was high, indicating that the data was objective and reliable (Table S3). The packages ggplot2 (version 3.4.3, Wickham 2016), jtools (version 2.2.2, Long 2022) and cowplot (version 1.1.1, Wilke 2020) were used for plotting graphs. To enhance reproducibility, we used a ‘co-pilot’ system where a co-author checked the lead author’s data processing and data analysis code (Reimer et al. 2019).

Pairwise relationships between behavioural measures, as hypothesised and outlined in Fig. 1, were assessed using correlation coefficient via the cor function from the stats package (R Core Team 2022). This approach allowed us to assess the strength and direction of linear relationships between each pair of variables. We checked the homogeneity of the variance by plotting the residuals against fitted values, and checked the normality of the residuals through a QQ plot. Assumptions were violated so we used Spearman rank correlation coefficients. As we also predicted that some measures would not correlate with each other, we calculated Bayes Factors (BF) instead of p-values using the correlationBF function in the package BayesFactor (version 0.9.12–4.6, Morey and Rouder 2023), on rank transformed data, with the default prior width. A BF > 1 supports the alternative hypothesis, whereas a BF < 1 supports of the null hypothesis. A BF around 1 indicates inconclusive evidence. The size of the BF indicates the strength of evidence: anecdotal (BF range between 1/3 − 1; or between 1 and 3), moderate (BF range between 1/10 − 1/3; or between 3 and 10), strong (BF range between 1/30 − 1/10; or between 10 and 30), very strong (BF range between 1/100 − 1/30; or between 30 and 100) or extreme (BF < 1/100; or > 100) (Schönbrodt and Wagenmakers 2018).

Missing values in each task—for example, when an individual interacted with the apparatus but failed to detour, making it impossible to calculate detour latency—were replaced with the time the individual spent in the test box. This value corresponds to the maximum trial duration minus the time spent in the start box. We replaced values with this maximum duration for *n* = 6 birds in the detour task (out of 99 participants) and *n* = 17 birds in the stop-change task (out of 98 participants). To assess robustness, we also reran the Spearman correlations excluding these individuals; the results are reported in Table S4.

For the main analyses, we pooled data across both species. We explore the effect of species on our ‘going’ and ‘stopping’ measures in another study (Troisi et al. 2024). However, for completeness, we also examined whether correlations differed between herring gulls and lesser black-backed gulls. Overall, species differences were minimal: only one of the 13 correlations differed significantly between species, and this was observed with only one of the two methods used to compare correlations (Table S5).

## Results

87 individuals participated in all three tasks (see Table S6 for an overview of individual participation in all three tasks, *n* = 33 herring gulls, *n* = 54 Lesser black-backed gulls).

### Descriptive statistics

#### Measures of ‘going’

On average, birds took 2.58 s to interact with the apparatus after leaving the start food area in the detour barrier task (range: 0.07–18.54 s, sd = 3.26; Figure S2.A), 4.34 s to interact with the apparatus after leaving the start food area in the thwarting task (range: 0.57–16.47 s, sd = 3.27; Figure S2.A), and 2.97 s to trigger the infrared beam in the stop-change task (range: 0.25–18.17 s, sd = 3.40; Figure S2.A).

#### Measures Of ‘stopping’

On average, it took birds 21.06 s to detour after leaving the start food (range: 0.72–137.89 s, sd = 23.47; Figure S2.B), and birds interacted for 10.71 s with the barrier in the detour barrier task (range: 0–58.96 s, sd = 10.99, 14 birds did not interact with the barrier; Figure S2.C). In the thwarting task, on average birds interacted with the food bowl for 25.76 s (range: 1.25–83.42 s, sd = 17.98; Figure S2.C). Finally, in the stop-change task, it took them on average 9.36 s to change direction (range: 0.50–99.65 s, sd = 17.71; Figure S2.B), and they were on average 50.26 pixels away from the ’old’ location (range: 0.48–198.44 pixels, sd = 46.21).

### Between- and within-task correlations

**Fig. 4:**
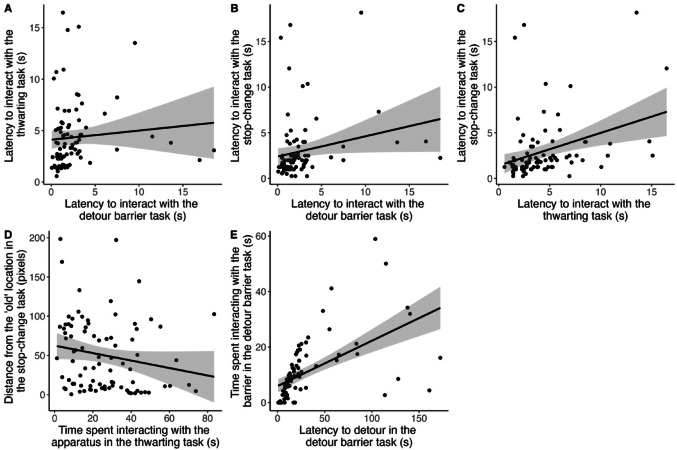
Correlations between the different variables where we found moderate to extreme support: (**A**) relationship between the latency to interact with the detour task and the thwarting task (measures of ‘going’), (**B**) relationship between the latency to interact with the detour task and the stop-change task (measures of ‘going’), (**C**) relationship between the latency to interact with the thwarting task and the stop-change task (measures of ‘going’), (**D**) relationship between the time spent interacting with the apparatus in the thwarting task and the distance to the ‘old’ location in that stop-change task (measures of ‘stopping’), (**E**) relationship between the latency to detour in the detour barrier task, and the time spent interacting with that barrier in the same task (measures of ‘stopping’). The black line shows the linear regression between the two variables, and the grey area shows the 95% confidence interval

We found strong to extreme evidence of positive correlations between our measures of ‘going’ (latencies to interact: between the detour barrier and thwarting tasks: *r* = 0.301, BF = 10.65, Fig. 4.A; between the detour barrier and the stop-change tasks: *r* = 0.393, BF = 195.92, Fig. 4.B; between the thwarting and stop-change tasks: *r* = 0.443, BF = 943.23, Fig. 4.C).

For measures of ‘stopping’, we found moderate support for a negative correlation between time spent interacting with the thwarting task and the distance to the ‘old’ location in the stop-change task (*r* = −0.248, BF = 3.09; see also Fig. 4.D; Table 2). On average, birds that spent a lot of time pecking at the apparatus in the thwarting task, got closer to the ‘old’ location in the stop-change task compared with individuals that pecked less. The other Bayesian tests provided anecdotal to moderate support for the null hypothesis (Table 2).

Finally, for the detour barrier and stop-change tasks, we had two measures of ‘stopping’. As can be seen in Table 2, we found extreme support for within-task correlations in the detour barrier task (Fig. 4.E) but moderate support for the null in stop-change task.

In the detour barrier task, the measure of ‘going’ and ‘stopping’ are the exact same (detour latency) if the individual did not interact with the barrier. We rerun the analyses after excluding those individuals (see Supplementary Materials, Table S2). Excluding these individuals (*n* = 14) did not influence the r values, but it did influence the Bayes factors (overall reduced support for the null hypotheses).

While including all individuals that participated allowed us to have greater discriminative power, gently pushing individuals into the test box (if they did not enter voluntarily) could have influenced the birds’ behaviour. We therefore repeated the analysis including only those individuals that entered the test box voluntarily in all three tests (*n* = 52). The results are presented in the supplementary material (Table S7). Similarly to the previous comparison, the r values changed very little, but the Bayes factors decreased.

**Table 2:** Correlation matrix showing the correlation coefficient and Bayes factor (in brackets) between the different behavioural measures of ‘stopping’ (*n* = 87). Results with moderate to extreme support for the alternative hypothesis are shown in bold regular; results with moderate support for the null hypothesis are shown in bold italic with a grey background

	Detour barrier: Latency to detour	Detour barrier: time spent interacting with the barrier	Thwarting: time spent interacting with the apparatus	Stop-change: latency to change
Detour barrier: time spent interacting with the barrier	**0.788 (> 1000)**			
Thwarting: time spent interacting with the apparatus	−0.147 (0.588)	***0.029 (0.254)***		
Stop-change: latency to change	0.181 (0.919)	***−0.005 (0.246)***	***−0.080 (0.317)***	
Stop-change: minimum distance to old location	***0.083 (0.323)***	***−0.055 (0.277)***	**−0.248 (3.09)**	***−0.087 (0.333)***

## Discussion

### Overview and predictions

The aim of Part 2 of this study was to investigate whether different measures of response inhibition, obtained across three different tasks, were related to each other, using a conceptual framework introduced in Part 1 (Troisi et al. 2026). Given that all tasks involved a similar ‘go stimulus’ (a food reward) and initial discrete action (approaching the food), we predicted that measures of ‘going’ would correlate across tasks. In contrast, we predicted less overlap between the measures of ‘stopping’ because the tasks differed (at least partly) in terms of the ‘stop stimuli’, the relative timing of the ‘go’ and ‘stop’ stimuli, and the type of action that had to be inhibited. Depending on the task feature of interest, in terms of between-tasks correlations, we predicted positive correlations between measures in tasks that shared the same type of stop-stimulus, the relative timing of the stimuli, and the same type of action to be inhibited (see Fig. 1). We also predicted a lack of correlations between measures in tasks that shared different task features (see Fig. 1). Our results showed positive correlations between all three measures of ‘going’ and some correlations between measures of ‘stopping’. However, not all predictions based on the theoretical framework that we developed in Part 1 were confirmed. Overall, our results show that inhibiting an action is not a single, unidimensional psychological ability, consistent with previous work in human cognitive psychology, neuroscience, and animal cognition (Bari and Robbins 2013; Beran 2015; Diamond 2013).

### Consistent correlations in the ‘going’ measures

As predicted, we found strong to extreme evidence for positive correlations between all three ‘going’ measures. This suggests that individual differences in approach behaviour are consistent across our tasks. These correlations may reflect stable traits such as cognitive processing speed (e.g. food detection or decision making), stress, walking speed or general motivation for food (Schubiger et al. 2020). Importantly, the correlations remained even when excluding individuals who were gently pushed into the test box (33 of the 87 birds that participated in all three tasks), although the strength of evidence (Bayes Factor) decreased slightly. This suggests that the consistency in ‘going’ behaviour is not solely due to motivation to participate in the tests.

We find such correlations between the ‘go’ measures in our three tasks despite small differences in task design. For instance, the ‘go stimulus’ (food reward) was further away in the stop-change task than in the other two tasks. In the detour barrier task, individuals also received previous experience in accessing food behind opaque barriers. In our study, we found that individuals which were fast in interacting with the detour barrier task were also fast in interacting with the other two tasks in which they had no prior training, suggesting that the training had little impact on the ‘go’ response of the detour barrier task. The results could also suggest that there are carry-over effects and that training had not only an effect on the ’go’ response in the detour task but also on the ‘go’ response in the other tasks.

Tasks were also performed in a fixed order (detour barrier first, then thwarting, then stop-change task). Habituation to the test box could have affected correlations between our ‘go’ measures if individuals habituate at different rates. Indeed, individuals with different coping styles will perform differently under standardised conditions (e.g. Mazza et al. 2019, 2020). However, given that we found strong to extreme support for positive correlations between our three ‘go’ measures, we find no evidence that habituation effects disrupted performance. This suggests that either all individuals habituated at a similar rate, or that variation in habituation did not meaningfully affect performance on the ‘go’ measures.

Overall, the strong consistency in ‘go’ responses supports the idea that the ‘go’ runner component of our framework captures a general behavioural tendency that is robust across task contexts.

### Mixed evidence for ‘stopping’ measures

**Fig. 5:**
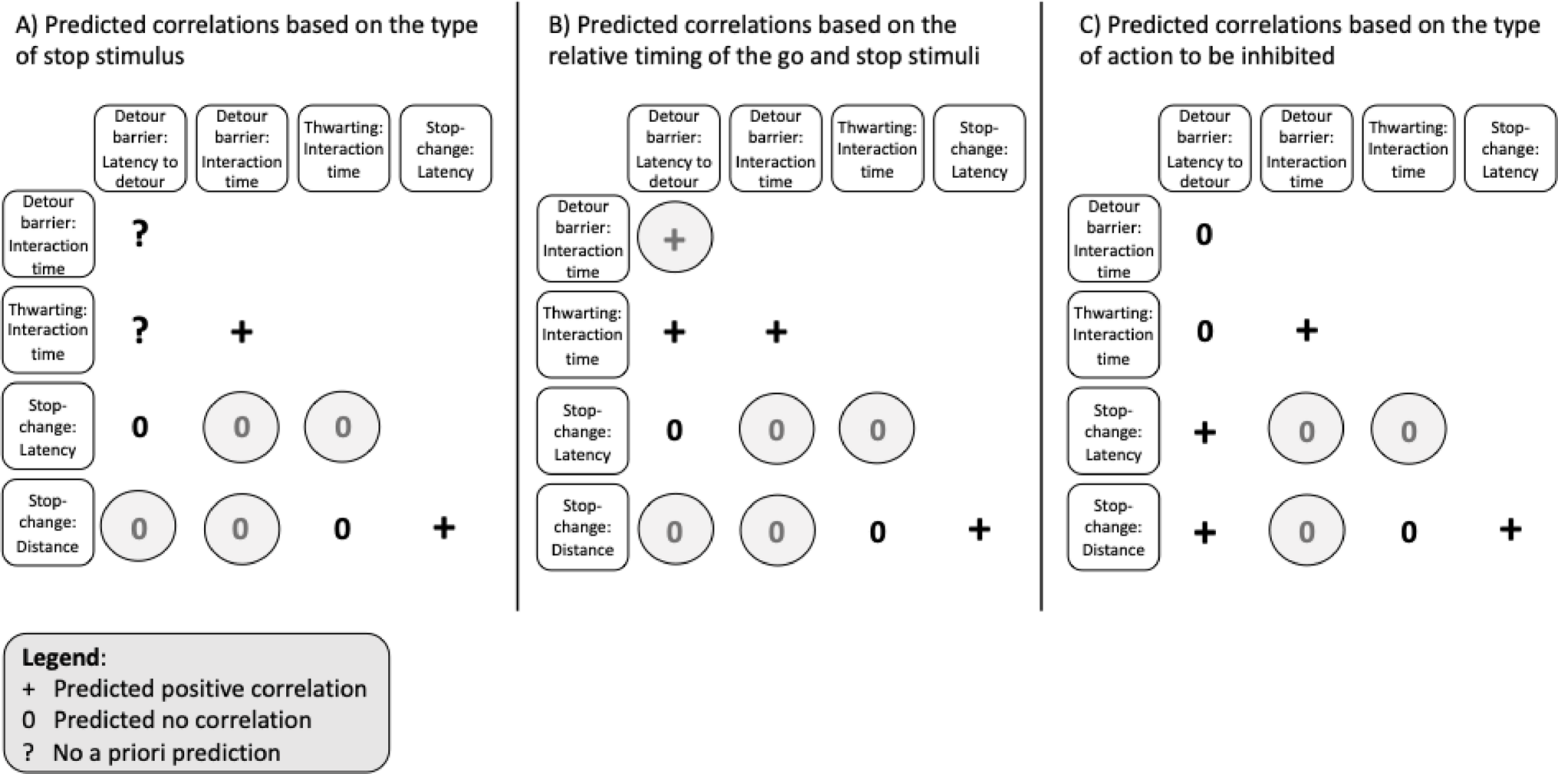
Predicted correlations between different measures of ‘stopping’ based on Fig. 1. Grey circles represent predictions supported by our results

In contrast to the consistent ‘go’ correlations, we found limited support for predicted correlations between ‘stop’ measures (Fig. 5). Of the predicted positive correlations based on task features, none were supported. We had predicted correlations between the latency to detour in the detour barrier task and (1) the time spent interacting with the thwarting task (based on the relative timing of the ‘go’ and ‘stop’ stimuli), (2) the latency to change direction in the stop-change task, and (3) the distance to the ‘old’ location in the stop-change task ((2) and (3) are both based on the type of action to be inhibited, see Figs. 1 and 5). In the latency to detour the barrier in the detour barrier task, and in the measure of the distance to the ‘old’ location in the stop-change task, it is difficult to disentangle the ‘go’ and ‘stop’ processes. This lack of purity in the measures could partly explain the observed lack of positive correlation (Troisi et al. 2026).

We also expected a correlation between the duration of interaction with the barrier in the detour barrier task and with the covered food bowl in the thwarting task. This is based on the overlap of all three task features highlighted in Fig. 1. While van Horik et al. (2018) found such a correlation in pheasant chicks, neither our study nor Garnham et al. (2022) replicated this finding. There are some differences that may explain the inconsistency between studies. First, in our study the individuals had gained some prior experience with transparent objects and navigating around them in their home enclosure (prior to testing). Similarly, individuals in Garnham et al.‘s (2022) study, who worked on red junglefowl (*Gallus gallus*), also had considerable prior experience with transparent objects, whereas the individuals in van Horik et al.‘s (2018) study did not. This relevant prior experience may have reduced their tendency to persistently peck at the barrier and allowed them to navigate around the barrier more effectively (Stow et al. 2018; van Horik et al. 2018), reducing variability (and therefore lowering correlations with other measures). Second, the thwarting task in our study included a free piece of fish on the cover of the food bowl, a feature absent in the detour barrier task (or in the other studies). While this piece of food was placed to initiate a go response (pecking) that would then need to be inhibited, this piece of food could have acted as a partial reinforcement, potentially influencing the pecking behaviour in the thwarting task.

Based on the Bayesian logic, and on the lack of overlap in the type of ‘stop stimulus’, the relative timing of the ‘go’ and ‘stop’ stimuli and the type of action that has to be stopped, we had predicted a lack of correlation between (1) the time spent interacting with the barrier in the detour barrier task and the latency to change direction in the stop-change task, (2) the time spent interacting with the barrier in the detour barrier task and the distance to the ‘old’ location in the stop-change task, (3) the time spent interacting with the cover in the thwarting task and the latency to change direction in the stop-change task, and (4) the time spent interacting with the cover in the thwarting task and the distance to the ‘old’ location in the stop-change task. Based on both the lack of overlap in the type of ‘stop stimulus’ and the relative timing of the ‘go’ and ‘stop’ stimuli, we had predicted a lack of correlation between (5) the latency to detour in the detour barrier task and the latency to change direction in the stop-change task, and (6) the latency to detour in the detour barrier task and the distance to the ‘old’ location in the stop-change task. Based only on the different action to be inhibited we had also predicted an absence of correlation between (7) the latency to detour in the detour barrier task and the time spent interacting in the thwarting task. We did find support for the absence of correlations between predictions 1–3 and 6, which partially supports our framework based on task features (Fig. 5). However, we found moderate support for a negative correlation between time spent interacting with the apparatus in the thwarting task and the minimum distance to the ‘old’ location in the stop-change task (prediction 4; Fig. 5). This was an unexpected result as the two measures do not have a similar ‘stop stimulus’, a similar timing between the ‘go’ and ‘stop’ stimuli, nor a similar action to be inhibited (see Fig. 5A, B, C). This suggests that another task feature might play a key role in predicting individual response to ‘stop’ stimuli.

Despite task differences such correlation between the thwarting and stop-change task measures could reflect a general inhibitory control mechanism (Robbins et al. 2012). In our framework, not persisting on the thwarting task is equivalent to “stopping”, but, at a neurological level, whether stopping in order to do nothing is similar to active motor inhibition is still debated (Hervault et al. 2021). Our results suggest that there is partial overlap between those two types of measures. Alternatively, the correlation observed might not be related to the ‘stop’ runner directly, since the ‘stop’ and ‘go’ runners are difficult to disentangle in those measures. Specifically, in the thwarting task, longer time spent pecking also reflects a race between a strong ‘go’ response and a ‘stop’ response (Troisi et al. 2026). In the stop-change task, if the duration of the ‘stop runner’ is held constant, birds that initially ran faster would naturally end up closer to the old location. Hence those measures might instead reflect an overlap in the ‘go runner’ (Carere and Locurto 2011; Dougherty and Guillette 2018; Sih and Del Giudice 2012).

We found that individuals that pecked more in the thwarting task also approached the unrewarded location more closely in the stop-change task. However, the relationship between distance to the ‘old’ location in the stop-change task and time spent pecking was not observed in the detour barrier task, suggesting that the correlation may not reflect a general inhibitory trait. Our two measures might instead reflect exploratory behaviour since both measures were found to correlate with information gathering behaviour in a novel environment task, but such relationship was not found with measures in the detour task (Vanhulle 2024).

### Within-Task consistency and measure validity

Given that measures within the same task would have either the same type of ‘stop stimulus’, relative timing of the ‘go’ and ‘stop’ stimuli or type of action that has to be stopped, we predicted that measures within the same task should correlate. In contrast to the between-task analyses, we found some within-task positive correlations. In the detour barrier task, there was extreme support for consistent relationships between the latency to detour and the time spent interacting with the barrier. For the stop-change task, our initial analysis did not show evidence for a correlation between the latency to change direction and the minimum distance to the ‘old’ location (Table 2), nor did our analysis when only including birds that entered the box voluntarily (Table S7). However, when we omitted individuals that never changed direction from the analysis (and therefore never approached the rewarded location), we do find extreme evidence for a correlation between these two measures (Table S4), with individuals that, on average, took longer to change direction being those with the smallest distance to the ‘old’ location. The lack of consistency in our findings also suggest that our results are not robust to changes in the analysis, potentially due to runner purity issues mentioned above, and further work on understanding the relationship between different measures of the stop-change task is required.

Since individuals that never changed direction were given a maximum score for a behaviour that never occurred, including such individuals weakened the correlation. When those individuals are excluded, our results (partially) mirror those of Garnham et al. (2022) and Meier et al. (2017), who found correlations between different measures in the detour and stop-change tasks, respectively. We are aware of only one other study using multiple measures within similar tasks to ours. Using problem-solving tasks that also involve a transparent component, van Horik and Madden (2016) found that individuals that pecked more frequently at the task were also more successful in obtaining a reward in the same task. This finding further highlights measure consistency within tasks. In our study, we did not use a measure of ‘success’, so our results are difficult to compare to those of van Horik and Madden (2016).

While such consistency within tasks is indicative of internal reliability, it is important to note that the within-task measures were not completely independent. That is, the latency to detour is likely to increase the longer an individual pecks at the barrier. Similarly, an individual that stops and changes quickly is also more likely to remain further away from the old location than an individual that stops and changes slowly (assuming that they initially ran at the same speed).

These examples highlight a broader issue: many within-task measures are functionally or temporally linked, which can artificially inflate correlations. This lack of independence complicates interpretation and underscores the need for caution when using such measures as distinct indicators of inhibitory control. Future studies should aim to design tasks and metrics that minimise such overlap, or statistically account for it during analysis.

### Revisiting the framework: what matters most?

In support of our framework, we found evidence for some of the predicted absences of correlations between our ‘stopping’ measures (Fig. 5). Those supported absences of correlations were predicted based on either the lack of similarity between all three task features or based on the lack of similarity between the ‘stop’ stimuli and the relative timing of the ‘go’ and ‘stop’ stimuli. Similarly to what Montalbano et al. (2020) found, these results suggest that the type of action to be inhibited may not matter as much when predicting correlations between tasks. Our results also highlight that more task feature similarities may be necessary for stronger correlations between measures. In fact, where we predicted a lack of correlation based on only one task feature, we did not find support for the lack of correlations (Fig. 5).

Our framework predicted that positive correlations in ‘stopping’ would depend on overlap in type of ‘stop’ stimulus, the relative timing of the ‘go’ and ‘stop’ stimuli, or the type of action to be stopped. The absence of predicted positive correlations suggests that these features may not be the primary drivers of individual differences in inhibition. Instead, other factors—such as motivation, frustration tolerance, or task-specific learning—may play a larger role. This could explain why multiple other studies investigating the overlap between tasks purported to measure the inhibition of discrete actions have found mixed results, both in terms of temporal and contextual repeatability (Ashton et al. 2018, 2022; Bray et al. 2014; Brucks et al. 2017; Davidson et al. 2022; Fagnani et al. 2016; Garnham et al. 2022; Marshall-Pescini et al. 2015; McCallum and Shaw 2023; Shaw 2017; Troisi et al. 2021; van Horik et al. 2018; Vernouillet et al. 2018).

Montalbano et al. (2020), who studied guppies (*Poecilia reticulata*), found a relationship between the inhibition of a repetitive action in a thwarting task (using the number of attacks at a tube as a measure) and a discrete action in a detour cylinder task (using the number of successful trials – without touching the cylinder – as a measure; note that this may be a measure of learning rather than response inhibition per se, see e.g., Willcox et al. 2025). According to our conceptual framework (Fig. 2; see also Part 1 of this study: Troisi et al. 2026), such a correlation could be due to the similarity of the ‘stop stimuli’ and the relative timing of the ‘go’ and ‘stop’ stimuli. But if the type of ‘stop stimulus’ and the relative timing of the ‘go’ and ‘stop’ stimuli were important, we should have observed correlations (1) between the time spent interacting with the barrier in the detour barrier task and the time spent interacting with the apparatus in the thwarting task, and (2) between the latency to detour in the detour barrier task and the time spent interacting with the apparatus in the thwarting task. We did not observe such correlations (Fig. 5), further indicating that other factors might be at play.

A possible explanation for the absence of expected positive correlations is the different sensitivities to external or internal factors in the tasks used. For instance, the thwarting task may be particularly sensitive to motivational differences, as it involves visible but inaccessible food. Unpublished findings from our lab suggest that birds that pecked longer in a thwarting task also ate more when food was available (Verbruggen et al. 2019). Moreover, this thwarting task is also used to measure frustration-induced motivation (Haskell et al. 2000). Similarly, the latency to detour in the detour barrier task and the distance measure of the stop-change task may conflate ‘stopping’ with ‘going’, making it difficult to isolate pure inhibitory control (Troisi et al. 2026). Although note that Meier et al. (2017) did not find a significant relationship between the minimum distance to the ‘old’ location and their motivation measure in the stop-change task with pheasants (*Phasianus colchicus*). These complexities highlight the challenge of designing tasks that cleanly measure inhibition. Future research in identifying the sensitives of each task will be very beneficial in developing the framework and identifying key factors influencing behaviour on tasks purporting to measure response inhibition.

Another possible explanation for the absence of expected positive correlations is that some measures of response inhibition were less reliable than others. Although we measured several components of response inhibition, we had only one trial per task. Determining the reliability and repeatability of cognitive measures requires multiple trials. However, task performance is strongly influenced by learning, including response inhibition learning (Willcox et al. 2025), which in turn could influence the measure of repeatability. Indeed, in detour tasks, individuals tend to improve and become faster over trials (reviewed in Kabadayi et al. 2018). To minimise learning effects, one could, for example, introduce different types of barriers during the detour barrier task (Davidson et al. 2022; Dewulf et al. 2025; McCallum and Shaw 2023; Sollis et al. 2023), different bowls and covers in the thwarting task, or different locations in the stop-change task. Estimating this temporal reliability would be crucial for the framework. Nevertheless, the problem remains that the consistency observed across trials may be due to factors unrelated to response inhibition.

So, what matters most? In our data, the type of action to be stopped was the least predictive of individual responses across tasks. The absence of the expected positive correlations further suggests that other task features, likely linked to the ‘go’ rather than the ‘stop’ runner, may play a larger role. More research is clearly needed to clarify how different task features shape responses.

### Limitations and future directions

In our study, we trained our gulls to detour opaque barriers and provided them with experience transparent barriers (although they did not have experience with food directly placed behind transparent barriers prior to the test). We added this prior training since some studies found that after training with opaque barriers, individuals perform better than without this training (Santos et al. 1999; Vernouillet et al. 2018) and there is evidence that experience of transparent barriers leads to learning about transparency (van Horik et al. 2018). We recommend using such prior experience to increase the likelihood of measuring response inhibition during the test, rather than learning (Willcox et al. 2025).

Our study focused on juvenile gulls, and it remains unclear whether the observed patterns would hold in adults. Different pathways involved in response inhibition in different task contexts might develop at different ages (Kabadayi et al. 2017). Environmental and/or species-specific factors may influence the development of response inhibition, and may only be detected once response inhibition has fully developed (e.g. Miller et al. 2020; Savaşçı et al. 2021; Vinogradov et al. 2022). We do not know the development of response inhibition in gulls, and as such, it would be interesting to test whether the predictions made by the framework, or the correlations found in our study, also hold when testing adults.

Additionally, we tested two closely related species, which show great level of behavioural flexibility at the species and individual levels. Our two species differ in key migratory and foraging strategies which could be related to differences in response inhibition (Troisi et al. 2024). While such behavioural differences could impact mean levels of response inhibition (which we explore in a separate study: Troisi et al. 2024), we suspected that it would not influence the relationship between measures of response inhibition in different tasks that we tested here. Our results suggest that this assumption was true, and support the generalisability of our findings within this ecological context.

## Conclusion

In conclusion, our study provides nuanced insights into the multifaceted nature of response inhibition in two gull species across three tasks. While ‘go’ measures were consistent across tasks, ‘stop’ measures were more variable and task specific. Our study highlights how different tasks and measures quantify different aspects of response inhibition, and therefore the importance of task design and the need for caution when interpreting inhibitory control as a stable trait.

By applying a conceptual framework and testing specific predictions with empirical data, we provide a more nuanced understanding of response inhibition in animals. Given tasks impurity, we were unable to determine a single best approach to measure response inhibition, as all measures are confounded by different task-specific and intrinsic factors that influence response inhibition. Future research should continue to refine task designs and explore the interplay of cognitive and non-cognitive factors driving inhibitory performance.

## Supplementary Information

Below is the link to the electronic supplementary material.

Supplementary Material 1

## Data Availability

Data and R Code are available on OSF: https://osf.io/jbe4q/?view\_only=cd763b15f4b649fb80e520fea326f0a3.
