# Supplementary material for: Unpacking response Inhibition in animals – part 2: an empirical test
Source: Anim Cogn. 2026 Jan 28;29(1):22. doi: 10.1007/s10071-025-02033-1 (PMC12872641; doi:10.1007/s10071-025-02033-1)
Supplement: Supplementary file 1 — Supplementary Material 1 [file 10071_2025_2033_MOESM1_ESM.docx]

**Supplementary Material**

**Methods**

We report our methods following the MeRIT system (Nakagawa et al. 2023).

Egg collection. From May 2021 to June 2021, eggs were collected on the Belgian coast (De Panne, Oostende, Blankenberge, Zeebrugge, Knokke) by the Agency for Nature and Forests (ANB) and the Wildlife Rescue Centre (WRC) Ostend, who are authorised to remove gull eggs along the Belgium coast for various reasons. Those eggs were brought to the WRC on the day of collection. Upon arrival, the eggs were weighed, measured, and photographed before incubating them. This was done until we reached our target sample size of 120 (Table S1).

Species was initially inferred from nest structure or parent species ID, for equal splitting between the conditions. This resulted in a total of 540 herring and lesser black-backed gull eggs collected from 205 nests. We only incubated a subset of these to obtain 120 subjects in our study.

Egg incubation**.** Upon arrival, CAT, AV and RA weighed, measured and photographed the eggs before placing them in incubators (OVA-Easy Advance; Brinsea; temperature: 37.5°C; humidity until pipping: 45%). For three-egg clutches, only the two largest ones (based on volume) were incubated. Eggs were checked twice a day (around 10:00 and 16:00) for signs of pipping. Pipping eggs were transferred to an incubator with higher humidity for hatching (OVA-Easy Advance; Brinsea; temperature: 37.5°C; humidity 70%). Due to technical issues with the incubators, initial mortality was high and additional eggs were incubated until we reached our target sample size.

Chick rearing. CAT, AV, RA, LL, FV and Michiel Cattrysse (MC) reared 120 chicks under standardised conditions. In line with the STRANGE framework, an overview of our sample size is provided in Table S1 (Webster and Rutz 2020). Once hatched, CAT, AV and RA gave chicks a unique combination of coloured rings. For the first 4 days of life, chicks were kept in indoor enclosures (50 x 50 cm), with a heating plate (brand: Comfort). During this time, we hand fed them with smelt, with added vitamins (Akwavit™) and water, 4 times a day. Once chicks were 5 days old, and if they weighed more than 60g, they were placed in outside enclosures (1.95 x 5.50 m, covered with pebbles, except for a 1 m long concrete area at the entrance). If they were 5 days old but weighed less than 60g, they were kept in the indoor enclosures until they reached the target weight. This was based on prior work from the Wildlife Rescue Centre in Oostende.

Once outside, chicks were fed mixture of fish (~25%) and puppy food pellets (28.67% protein) soaked in water (~75%) with added vitamins (Akwavit™). Food was delivered in two feeding stations (~1 x 1 m) and placed inside the home enclosure, on the concrete area of the enclosure (which was the only part of the enclosure with a 2m high roof), with a barrier placed at the entrance of the feeding station (Figure S1). Water was available in the food bowls. Once a group was complete, food was placed in front of the barrier for the first two days. Subsequently and for the remaining of the experiment, food was placed behind the barriers, hence providing individual with the experience of detouring opaque barriers. Birds were fed 3 times a day, following the schedule described in the early-life manipulation.

Figure S1: Outdoors home enclosure with feeding station with opaque barriers in the foreground, and three sets of transparent barriers in the background. Note: not pictured here, but opaque barriers also had coloured tape around their edges, in a similar way to the transparent barriers.

**Early-life manipulation.** Four outdoor enclosures of 15 chicks (n = 60) were assigned to a ‘predictable food’ treatment, and four outdoor enclosures of 15 chicks (n = 60) were assigned to an ‘unpredictable food’ treatment. Siblings were split between the two treatments to control for genetic and pre-hatching environment. Species were also split between the two treatments to get an equivalent sample size of each species in each treatment (Table S1). We only knew the sex after the allocation to the treatment, so sex was randomly distributed across treatment (Table S1).

Initially, we aimed to manipulate food predictability for two weeks after group completion. Due to the aforementioned technical issues, we could only implement this scheme for seven days. In brief, time of feeding was either predictable (i.e., feeding was at the same time each day: 9:00, 13:00, 17:00) or unpredictable (i.e., feeding time varied between days: 8:00, 9:00 or 10:00; 12:00, 13:00 or 14:00; 17:00 or 18:00). Furthermore, we added a ‘frustration manipulation’ (Days 3-7 of the manipulation) during the morning and the evening feeding sessions for chicks in the unpredictable treatment: food that was placed in the enclosure was retrieved for 1 minute as soon as the first chick ate, before giving back the food to the chicks. This procedure was repeated three times during each session, after which the food was left in the enclosure.

We ran these two different treatments for a different study (Troisi et al. 2024). While the gulls were fed under two different feeding regimes, we did not expect the feeding regime to influence the relationship between different measures of response inhibition so collapsed the data.

**Behavioural tests**. CAT, AV, RA, FV, MC, and LL conducted the behavioural tests. SK and Lumi Vanhulle coded the videos and RA and CAT double coded 20% of the videos. FV extracted distance data in the stop-change task.
After testing, when individuals were between 25 and 39 days old, they were housed in a large flight cage (approximately 180 m^2^) for approximately four to six weeks (depending on the finishing time of the tests) and were subsequently released in the wild.

**Species ID and sex**. DNA sampling for species ID and sex were conducted by Viki Vandomme, using feather samples collected when chicks hatched, following Fridolfsson and Ellegren (1999). We used the NucleoSpin Tissue extraction kit, with primers 2550F/2718R targeting the CHD1W and CHD1Z introns. PCR amplification was carried out in a total volume of 10 µL. We used the DreamTaq Green PCR Master Mix (2X) from thermofisher. Instead of a touchdown polymerase chain reaction described in Fridolfsson and Ellegren (1999), we used a normal PCR, which followed this cycle: (1) initial denaturation for 3 min at 95°C, (2) 3-step cycling (x30) which included 30 sec of denaturation at 94°C, 30 s of annealing at 56°C, and 60s of extension at 72°C, and a (3) final extension for 10 min at 72°C. For individuals for which the species could not be identified using DNA methods (e.g., because there were not enough feathers available), RA confirmed the species by using morphological characteristics when individuals were ringed prior to release. The ringing was done by AV and RA. For individuals for which the sex could not be identified using DNA methods, Hans Matheve used a support vector machine classifier to predict individual’s sex. Using birds for which we knew the sex and had measurements of weight, tarsus length, wing length, and head length when they were ringed, we predicted the sex of the remaining individuals for each species separately (accuracy of prediction for LBBG: 0.956 [95% C.I. 0.876-0.991], p-value<0.001; n = 15; accuracy of prediction for HG: 0.915 [95% C.I. 0.796-0.976], p-value<0.001; n = 11). There was one individual for which we did not have molecular data for sex identification, and which died before it was ringed, so we could not sex it using the predictive model. RA and MC measured the tarsus length prior to the behavioural tests, using a calliper, while the gull was placed in their laps. Both the left and right tarsus were measured twice, and the average of all four measurements was used.

**Statistical analysis.** CAT conducted the analysis. Using a co-pilot system RA checked the data processing codes, while AV checked the analysis code.

**Sample description**

Table S1: Following Webster & Rutz (2020), this table provides a description of our sample size based on species, early-life environment, and sex (we were not able to get the sex of one individual).

|  | Predictable | Unpredictable |
| --- | --- | --- |
| HG | 22 | 24 |
| LBBG | 38 | 36 |

|  | Female | Male |
| --- | --- | --- |
| HG | 27 | 19 |
| LBBG | 34 | 39 |

**Analyses including only birds that interact with the barrier in the detour task**

For the detour task, we defined the latency to interact with the apparatus as the latency to interact with the barrier, or for those that did not interact with the barrier (n = 14) the latency to detour. The correlation between the latency to interact with the apparatus and the latency to detour may have been spuriously increased as a result (as for 14 birds this was the same measure). We therefore reran the correlation between all variables (n = 73), by excluding those birds. The results are reported below and the main results remain unchanged.

Table S2: Correlation matrix showing the correlation coefficient and Bayes factor between the different behavioural measures (n = 73). In bold are results supporting moderate to extreme evidence for the alternative hypothesis, and in bold and italic with a grey background are results supporting moderate evidence for the null hypothesis. In (A) are behavioural measures corresponding to the “go” component; in (B) are those corresponding to the “stopping” component.

| A. Going | Detour barrier: latency to interact with the task | Thwarting: Latency to interact with the task |
| --- | --- | --- |
| Thwarting: latency to interact with the task | **0.322 (16.61)** |  |
| Stop-change: latency to interact with the task | **0.330 (11.58)** | **0.484 (>1000)** |

| B. Stopping | Detour barrier: Latency to detour | Detour barrier: time spent interacting with the barrier | Thwarting: time spent interacting with the apparatus | Stop-change: latency to change |
| --- | --- | --- | --- | --- |
| Detour barrier: time spent interacting with the barrier | **0.714 (>1000)** |  |  |  |
| Thwarting: time spent interacting with the apparatus | -0.166 (0.666) | 0.126 (0.450) |  |  |
| Stop-change: latency to change | 0.247 (2.09) | ***0.009 (0.268)*** | -0.116 (0.416) |  |
| Stop-change: minimum distance to old location | 0.116 (0.416) | ***-0.004 (0.267)*** | **-0.322 (9.60)** | ***-0.011 (0.268)*** |

Compared to the results reported in the main text, when excluding those 14 birds results remain similar, except that we find (1) moderate rather than extreme evidence of a correlation between the latency to interact with the detour barrier task and the latency to interact with the stop-change task, (2) anecdotal rather than moderate evidence of an absence of correlation between the latency to detour in the detour barrier task and the minimum distance to the old location in the stop-change task, (3) between the time spent interacting with the barrier in the detour task and time spent interacting with the apparatus int the thwarting task, and (4) anecdotal rather than moderate evidence of an absence of correlation between the time spent interacting with the barrier in the thwarting task and the latency to change direction in the stop-change task.

**Inter-coder reliability**

Table S3**:** Table showing for each behavioural variable used in the analysis, the interclass correlation coefficient (ICC), its p-value, and the sample size of the comparison between the two ratters (i.e. number of videos that were double-coded in which the behaviour occurred)

| Task | Variable Inclusion | Measure | Sample size | ICC | p-value |
| --- | --- | --- | --- | --- | --- |
| Detour | Analysis | Time spent interacting with the barrier | 21 | 0.992 | <0.001 |
|  |  | Latency to exit | 24 | 0.999 | <0.001 |
|  |  | Latency to detour | 21 | 1.00 | <0.001 |
|  | Analysis and Data inclusion | Latency to interact with the barrier | 21 | 0.907 | <0.001 |
|  |  | Latency to interact with start food | 23 | 0.873 | <0.001 |
|  |  | Latency to leave the start food | 23 | 0.998 | <0.001 |
|  | Data inclusion | Latency to eat | 20 | 1 | <0.001 |
| Thwarting | Analysis | Time spent interacting with the apparatus | 20 | 0.971 | <0.001 |
|  |  | Latency to exit | 24 | 1.00 | <0.001 |
|  | Analysis and Data inclusion | Latency to interact with the apparatus | 20 | 0.962 | <0.001 |
|  |  | Latency to interact with start food | 23 | 0.998 | <0.001 |
|  |  | Latency to leave the start food | 23 | 1 | <0.001 |
|  | Data inclusion | Latency to eat | 17 | 0.998 | <0.001 |
| Stop-change | Analysis | Latency to exit | 23 | 0.997 | <0.001 |
|  |  | Latency to change | 15 | 0.993 | <0.001 |
|  | Analysis and Data inclusion | Latency to cross the IR beam | 18 | 0.989 | <0.001 |

**Analysis when we exclude individuals that do not detour (n = 6) and those who do not change direction n = 17)**

For latency to detour in the detour barrier task, and latency to change direction in the stop-change task, we have individuals with missing values (some individuals did not do those behaviour). In our initial analysis we replaced these values with the maximum possible latency: we did this for n = 6 for the latency to detour barrier (out of 99 birds that participated in the detour task), and n = 17 for the latency to change (out of 98 birds that participated in the stop-change task). Here we reran the correlation without those individuals:

Table S4: Correlation matrix showing the correlation coefficient and Bayes factor between the different behavioural measures when we exclude individuals that either do not detour in the detour barrier task or do not change direction on the stop-change task. In bold are results supporting moderate to extreme evidence for the alternative hypothesis, and in bold and italic with a grey background are results supporting moderate evidence for the null hypothesis.

|  | Detour barrier: Latency to detour | Detour barrier: time spent interacting with the barrier | Thwarting: time spent interacting with the apparatus | Stop-change: latency to change |
| --- | --- | --- | --- | --- |
| Detour barrier: time spent interacting with the barrier | **0.855 (>1000)**  **(was extreme evidence for the alternative hypothesis, and still is)** |  |  |  |
| Thwarting: time spent interacting with the apparatus | -0.134 (0.504) (was anecdotal evidence for the null hypothesis, and still is) |  |  |  |
| Stop-change: latency to change | 0.159 (0.610) (was anecdotal evidence for the null hypothesis, and still is) | ***0.077 (0.324) (was moderate evidence for the null, and still is)*** | ***0.033 (0.276) (was moderate evidence for the null, and still is)*** |  |
| Stop-change: minimum distance to old location | ***0.066 (0.296) (was moderate evidence for the null, and still is)*** |  |  | **-0.405 (0.285) (was moderate evidence for the null hypothesis, and changed to extreme evidence for the alternative)** |

Compared to the results reported in the main text, when excluding those birds results remain similar, except that we find extreme support for the alternative hypothesis rather than moderate evidence of a correlation between the latency to change direction in the stop-change task and the distance to the ‘old’ location in the stop-change task.

**Species differences**

Because we used two different species in our analysis we compared the correlations between the two species (n = 33 HG; n = 54 LBBG) by using two different methods for robustness: (1) comparing the spearman rank correlations’ 95% confidence intervals, and whether they overlap, and (2) the *cocor* package on rank transformed data (Diedenhofen 2012). The results are provided below, and show that there are no major differences in the relationship between the different variables in the two species, except for the correlation between the latency to detour and the time spent interacting with the thwarting task. But such difference between species only comes up using the *cocor* method, while the confidence intervals of the correlations for each species do overall, so we suggest that this difference is not robust.

Given the similarities in the correlations between species, we report in the main manuscript the two species combined.

Table S5: Correlation matrix for each species, including information about the overlap in confidence interval between species and the *cocor* comparison

| Response Inhibition | First Parameter | Second Parameter | Species | r | 95% CI | P value | Overlap of 95% CI | P value comparison (cocor) |
| --- | --- | --- | --- | --- | --- | --- | --- | --- |
| Go | Detour: latency to interact | Thwarting: latency to interact | HG | 0.93 | 0.87; 0.97 | <0.001 | Yes | 0.782 |
|  |  |  | LBBG | 0.89 | 0.82; 0.94 | <0.001 |  |  |
|  | Detour: latency to interact | Stop-change: latency to interact | HG | 0.20 | -0.17; 0.52 | 0.268 | Yes | 0.550 |
|  |  |  | LBBG | 0.33 | 0.06; 0.55 | 0.016 |  |  |
|  | Thwarting: latency to interact | Stop-change: latency to interact | HG | 0.18 | -0.19; 0.50 | 0.320 | Yes | 0.659 |
|  |  |  | LBBG | 0.30 | 0.02; 0.53 | 0.029 |  |  |
| Stop | Detour: latency to detour | Detour: time spent interacting | HG | 0.68 | 0.43; 0.83 | <0.001 | Yes | 0.161 |
|  |  |  | LBBG | 0.82 | 0.70; 0.89 | <0.001 |  |  |
|  | Detour: latency to detour | Thwarting: time spent interacting | HG | -0.47 | -0.71; -0.14 | 0.005 | Yes | **0.020** |
|  |  |  | LBBG | 0.02 | -0.25; 0.30 | 0.872 |  |  |
|  | Detour: latency to detour | Stop-change: latency to change | HG | 0.26 | -0.11; 0.56 | 0.149 | Yes | 0.356 |
|  |  |  | LBBG | 0.05 | -0.23; 0.32 | 0.718 |  |  |
|  | Detour: latency to detour | Stop-change: distance | HG | 0.23 | -0.13; 0.54 | 0.189 | Yes | 0.390 |
|  |  |  | LBBG | 0.04 | -0.24; 0.31 | 0.766 |  |  |
|  | Detour: time spent interacting | Thwarting: time spent interacting | HG | -0.21 | -0.52; 0.16 | 0.245 | Yes | 0.080 |
|  |  |  | LBBG | 0.19 | -0.09; 0.44 | 0.172 |  |  |
|  | Detour: time spent interacting | Stop-change: latency to change | HG | -0.19 | -0.51; 0.18 | 0.301 | Yes | 0.445 |
|  |  |  | LBBG | -0.01 | -0.29; 0.26 | 0.930 |  |  |
|  | Detour: time spent interacting | Stop-change: distance | HG | 0.05 | -0.31; 0.40 | 0.784 | Yes | 0.492 |
|  |  |  | LBBG | -0.11 | -0.37; 0.17 | 0.437 |  |  |
|  | Thwarting: time spent interacting | Stop-change: latency to change | HG | -0.19 | -0.51; 0.17 | 0.290 | Yes | 0.454 |
|  |  |  | LBBG | -0.02 | -0.29; 0.26 | 0.886 |  |  |
|  | Thwarting: time spent interacting | Stop-change: distance | HG | -0.13 | -0.46; 0.23 | 0.475 | Yes | 0.295 |
|  |  |  | LBBG | -0.35 | -0.57; - 0.09 | 0.009 |  |  |
|  | Stop-change: latency to change | Stop-change: distance | HG | 0.09 | -0.27; 0.43 | 0.629 | Yes | 0.223 |
|  |  |  | LBBG | -0.19 | -0.44; 0.09 | 0.168 |  |  |

**Participation**

Table S6: List of individuals that do not show specific behaviour of interest. Highlighted in red are the individuals which were excluded from the analysis (X: did not show a particular behaviour; NA: we do not have the data due to technical issues). 21 individuals did not interact with the detour task, 15 with the thwarting task, and 15 with the stop-change task. In the stop-change task we also lost data for 7 individuals due to technical issues. In total, we had to exclude 33 individuals from our initial analysis.

|  | Detour | | | | Thwarting | | | Stop change | | Excluded from correlation |
| --- | --- | --- | --- | --- | --- | --- | --- | --- | --- | --- |
|  | Did not interact with barrier | Did not detour | Did not eat | Excluded from detour | Did not interact with apparatus | Did not eat | Excluded from thwarting | Did not cross the IR beam (excluded from stop-change) | Did not change |  |
| Sample size | 37 | 22 | 28 | 21 | 17 | 31 | 15 | 15 (22 excluded when including NA) | 32 | 33 |

**Individual variation**


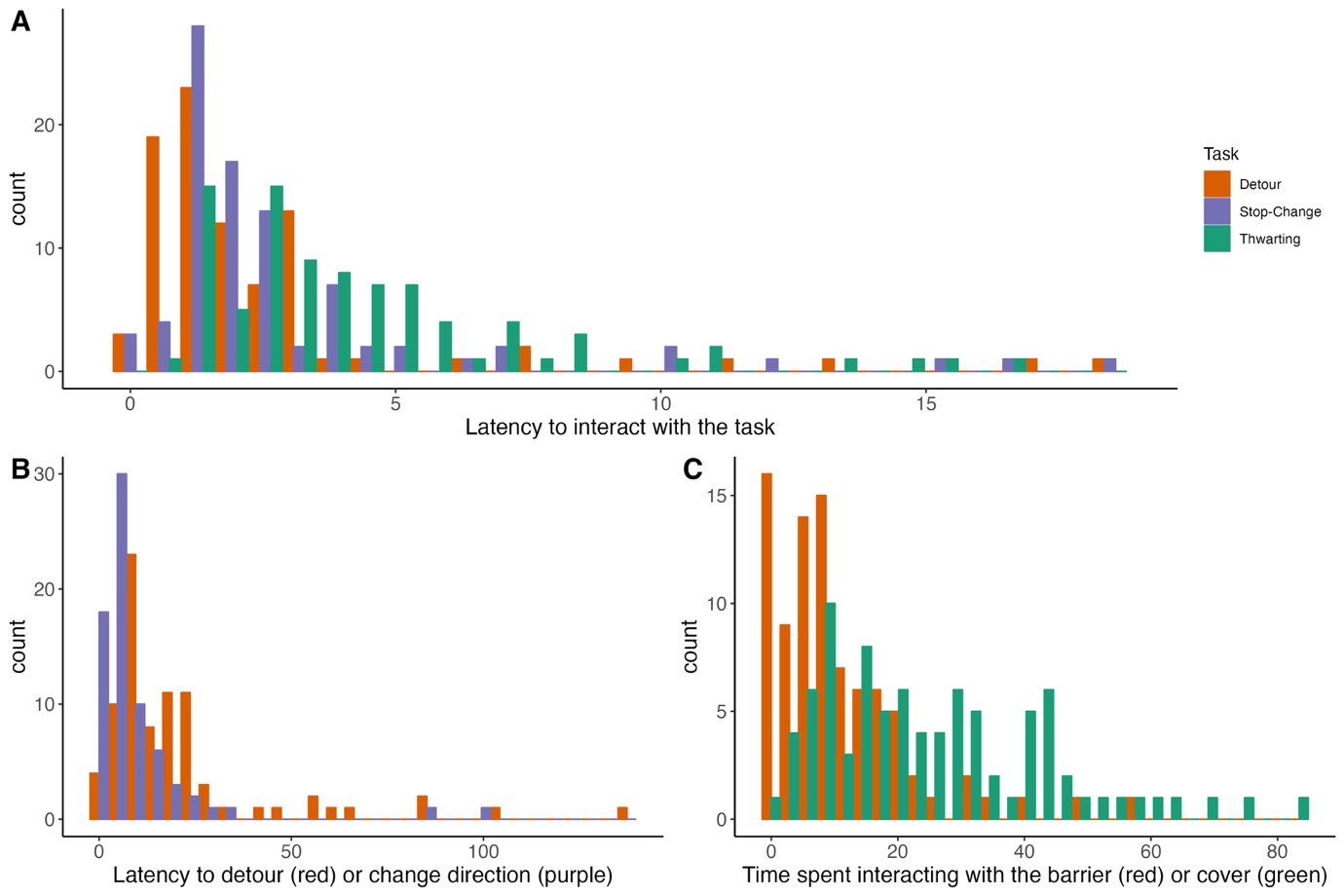


Figure S2: Histograms showing variation across all three tasks in the (A) ‘going’ measures (latency to interact with the detour (red), thwarting (green), and stop-change (purple) tasks), (B) measures related to inhibiting discrete actions (latency to detour in the detour task (red) and latency to change direction in stop-change task (purple), and (C) related to inhibiting repetitive actions (time spent interacting with the transparent barrier in the detour task (red), and transparent cover in the thwarting task (green)).

**Analyses including only birds that entered the test arena voluntarily (n = 52)**

Some individuals did not enter the test arena voluntarily, and this could impact the behaviour we measured, particularly those related to latency. We therefore repeated the analysis including only those individuals that entered the test box voluntarily in all three tests (n = 52, Table S7), to evaluate the robustness of our results by accounting for potential (lack of) motivation or fear.

Table S7: Correlation matrix showing the correlation coefficient and Bayes factor between the different behavioural measures (n = 52). In bold are results supporting moderate to extreme evidence for the alternative hypothesis, and in bold and italic with a grey background are results supporting moderate evidence for the null hypothesis. In (A) are behavioural measures corresponding to the “go” component; in (B) are those corresponding to the “stopping” component.

| A. Going | Detour barrier: latency to interact with the task | Thwarting: Latency to interact with the task |
| --- | --- | --- |
| Thwarting: latency to interact with the task | **0.358 (6.74)** |  |
| Stop-change: latency to interact the task | **0.384 (11.22)** | **0.473 (90.04)** |
|  |  |  |
|  |  |  |

| B. Stopping | Detour barrier: Latency to detour | Detour barrier: time spent interacting with the barrier | Thwarting: time spent interacting with the apparatus | Stop-change: latency to change |
| --- | --- | --- | --- | --- |
| Detour barrier: time spent interacting with the barrier | **0.859 (>1000)** |  |  |  |
| Thwarting: time spent interacting with the apparatus | 0.085 (0.368) | 0.297 (2.46) |  |  |
| Stop-change: latency to change | 0.121 (0.436) | ***0.011 (0.313)*** | ***-0.049 (0.328)*** |  |
| Stop-change: minimum distance to old location | 0.149 (0.518) | ***-0.036 (0.322)*** | -0.253 (1.37) | -0.284 (2.06) |

Compared to the results reported in the main text, when excluding those 35 birds that did not enter the test arena voluntarily, results remain similar except that we find (1) moderate rather than strong evidence for a positive correlation between the latency to interact with the detour barrier task and the latency to interact with the thwarting task, (2) strong rather than extreme evidence for a positive correlation between the latency to interact with the detour barrier task and the latency to interact with the stop-change task, (3) very strong rather than extreme evidence for a positive correlation between the latency to interact with the thwarting task and the latency to interact with the stop-change task, (4) anecdotal evidence for a positive correlation rather than moderate evidence of a lack of correlation between the latency to detour in the detour barrier task and the minimum distance to the old location in the stop-change task, (5) anecdotal evidence rather than moderate evidence of a lack of correlation between the time spent interacting with the barrier in the detour barrier task and time spent interacting with the apparatus in the thwarting task, (6) anecdotal evidence rather than moderate evidence for a negative correlation between the time spent interacting with the apparatus in the thwarting task and the minimum distance to the old location in the stop-change task, (7) anecdotal evidence for a negative correlation rather than moderate evidence of a lack of correlation between the latency to change direction in the stop-change task and the and the minimum distance to the old location in the stop-change task.

We decided to present in the main text the analysis with all participating birds, as this allows for more power, but we discuss in our discussion that our results are not all supported when we adjust the sample so should be interpreted with caution.

**References**

Diedenhofen, Birk. 2012. “Cocor: Comparing Correlations.” November 30. https://doi.org/10.32614/CRAN.package.cocor.

Fridolfsson, Anna-Karin, and Hans Ellegren. 1999. “A Simple and Universal Method for Molecular Sexing of Non-Ratite Birds.” *Journal of Avian Biology* 30 (1): 116. https://doi.org/10.2307/3677252.

Nakagawa, Shinichi, Edward R. Ivimey-Cook, Matthew J. Grainger, et al. 2023. “Method Reporting with Initials for Transparency (MeRIT) Promotes More Granularity and Accountability for Author Contributions.” *Nature Communications* 14 (1): 1788. https://doi.org/10.1038/s41467-023-37039-1.

Troisi, Camille A., Alizée Vernouillet, R Allaert, et al. 2024. *Beyond a Unitary Construct: Dissecting Response Inhibition in Two Bird Species - Part 2: Variation across Species*.

Webster, Michael M., and Christian Rutz. 2020. “How STRANGE Are Your Study Animals?” *Nature* 582: 337–40.
